# Wear Fast, Die Young: More Worn Teeth and Shorter Lives in Iberian Compared to Scottish Red Deer

**DOI:** 10.1371/journal.pone.0134788

**Published:** 2015-08-07

**Authors:** F. J. Pérez-Barbería, J. Carranza, C. Sánchez-Prieto

**Affiliations:** 1 The James Hutton Institute, Craigiebuckler, AB15 8QH, Aberdeen, Scotland, United Kingdom; 2 Ungulate Research Unit, CRCP, University of Córdoba, Córdoba, Spain; 3 Grupo PAIDI RNM118, Estación Biológica de Doñana, CSIC, Sevilla, 41092, Spain; 4 Department of Zoology, University of Granada, Granada, 18071, Spain; Monash University, AUSTRALIA

## Abstract

Teeth in Cervidae are permanent structures that are not replaceable or repairable; consequently their rate of wear, due to the grinding effect of food and dental attrition, affects their duration and can determine an animal's lifespan. Tooth wear is also a useful indicator of accumulative life energy investment in intake and mastication and their interactions with diet. Little is known regarding how natural and sexual selection operate on dental structures within a species in contrasting environments and how these relate to life history traits to explain differences in population rates of tooth wear and longevity. We hypothesised that populations under harsh environmental conditions should be selected for more hypsodont teeth while sexual selection may maintain similar sex differences within different populations. We investigated the patterns of tooth wear in males and females of Iberian red deer (*Cervus elaphus hispanicus*) in Southern Spain and Scottish red deer (*C*. *e*. *scoticus*) across Scotland, that occur in very different environments, using 10343 samples from legal hunting activities. We found higher rates of both incisor and molar wear in the Spanish compared to Scottish populations. However, Scottish red deer had larger incisors at emergence than Iberian red deer, whilst molars emerged at a similar size in both populations and sexes. Iberian and Scottish males had earlier tooth depletion than females, in support of a similar sexual selection process in both populations. However, whilst average lifespan for Iberian males was 4 years shorter than that for Iberian females and Scottish males, Scottish males only showed a reduction of 1 year in average lifespan with respect to Scottish females. More worn molars were associated with larger mandibles in both populations, suggesting that higher intake and/or greater investment in food comminution may have favoured increased body growth, before later loss of tooth efficiency due to severe wear. These results illustrate how independent selection in both subspecies, that diverged 11,700 years BP, has resulted in the evolution of different longevity, although sexual selection has maintained a similar pattern of relative sex differences in tooth depletion. This study opens interesting questions on optimal allocation in life history trade-offs and the independent evolution of allopatric populations.

## Introduction

Teeth are permanent structures that, once erupted, suffer a process of continuous degradation by the wearing effect of food comminution and attrition (i.e. mechanical forces from opposing teeth) and, in ungulates and some other mammals, they cannot be replaced or repaired across the animal’s life [1,2].

As ungulate teeth are not replaceable or repairable, their rate of wear is a useful indicator of accumulative life energy investment in intake and mastication and their interactions with diet, as the abrasive effect of forage, phytoliths and soil adhered to external surfaces differs between plants, their stage of maturity and environments [3,4]. Due to this accumulative effect, tooth wear provides a longitudinal view of the animal’s strategy to balance current intake and lifetime maintenance [5–7].

The two main functions of ungulate teeth are the cropping-shearing of grass swards or browse stands (intake) and the mechanical grinding of food to reduce its particle size prior to chemical reduction in the gut, adding new surface area on which enzymes can act more quickly (digestion) [2,8]. The first function is accomplished by the incisors and the second by premolars and molars, in both cases aided by the soft structures of the oral apparatus (lips, cheeks and tongue) [2,3].

A decrease in molar performance reduces the number of food particles produced by a mastication stroke, which has a detrimental effect on digestion [9]. A decrease in incisor performance has a detrimental effect on tooth shearing capability but also on bite size [2], as the width of the incisor arcade decreases with incisor wear [10–12]; this reduces efficiency in severing food, possibly affecting food selection and intake per unit of time [13–15]. There is evidence that severe tooth wear has a negative effect on fitness [6], and that senescence and lifespan are closely related to tooth duration [16].

Teeth have evolved to be adaptable to changing conditions, to cope with seasonal changes in plant quality and availability, and with changes in plant composition associated with animal movement, such as migrations. However, drastic shifts in flora, associated with paleoclimatic changes, have imposed selection pressures toward tooth morphologies (e.g. hypsodonty: high-crowned teeth) better adapted to the new conditions, [17]. It is unclear whether deer populations have evolved teeth adapted to a wide range of conditions, or if deer populations that live in contrasting habitats differ in tooth morphology [18,19]. Contrasting habitats differ in the type of forage available (for example, the proportion of graze and browse), as well as its abrasiveness and fibrousness (higher in arid environments), all of which affect tooth wear. If the teeth of different populations are adapted to cope with these different conditions, then one would expect to find differences in tooth morphology between populations: with more durable teeth in environments with highly fibrous and abrasive food [17,18]. Alternatively, differences in rates of tooth wear between populations would be expected to have repercussions for lifespan.

The effect of environmental conditions on tooth wear may not be equivalent in molars and incisors. Veiberg et al. [20] studied incisor and molar wear in red deer and moose (*Alces alces*) in several populations in Norway and found that inter-population effects differed between incisors and molars. In supposedly harsher conditions (more arid environment, less available energy density), molars experienced higher wear rates but this was not the case for incisors. They argued that scarce, low quality food might require more chewing in order to achieve the equivalent comminution of lower fibre diets, thus affecting molar wear, while at the same time the lower amount of available forage would explain a lower incisor wear. However, it remains unclear why low quality food is more demanding on comminution chewing than in cropping and severing biting, as well as its effects in the evolution of more hypsodont teeth and in longevity.

Teeth in male and female deer differ in life span [5,7] and size relative to body size [16,21]. These differences are the result of sexual selection acting on males and females, either because of sex-differences in diet selection [22] or in the optimal timing of tooth use relative to the sex-specific timing of growth and reproduction across lifetime [7,16].

Current red deer populations in Western Europe originated from postglacial colonization from the Iberian refugia during the Holocene [23]. Thus, Scottish and Iberian populations share common ancestors from the Würn last glacial maximum, ca. 12000 years ago. Divergence between both lineages took place when Scottish ancestors expanded to the north, while ancestors of modern Iberian red deer remained in the south and thus each experienced the differing Holocene climate and environmental changes in their respective ranges [24,25].

Here we investigate patterns of tooth wear in males and females of these two red deer populations, Iberian red deer and Scottish red deer, which now live in very contrasting environments, separated by 20° latitude. On the basis of existing literature regarding tooth wear in red deer and other ungulates, we may outline the following predictions:

(H_1_) increased molar and incisor wear rates in the Iberian population, as xerophilous Mediterranean flora is expected to be more abrasive than the boreal Palearctic ecozone Scottish flora.

(H_2_) more hypsodont molars and incisors (i.e. more durable) in the Iberian population in comparison with the Scottish one, as an adaptive compensation for higher wear rates as explained in H_1_;

(H_3_) higher tooth wear rate in males than in females, as males of highly polygynous species invest in more rapid growth and reproduction within a shorter schedule of reproductive life compared to females;

(H_4_) different wear pattern between incisors and molars across age, as molars and incisors perform different functions, and incisors may last longer as they do not suffer attrition wear (i.e. no upper incisors in Cervidae);

(H_5_) because tooth wear is mainly the result of the amount of food processed for maintenance, growth and reproduction, there should be a trade-off between attaining body size and preserving teeth, which may differ between both populations if maintenance costs, due for instance to thermoregulation, differ between locations.

Understanding differences in tooth wear between populations, sexes, and tooth type could help to reveal insights into the interactions between tooth function, evolution and sexual selection in contrasting environments.

## Materials and Methods

**Fig 1:**
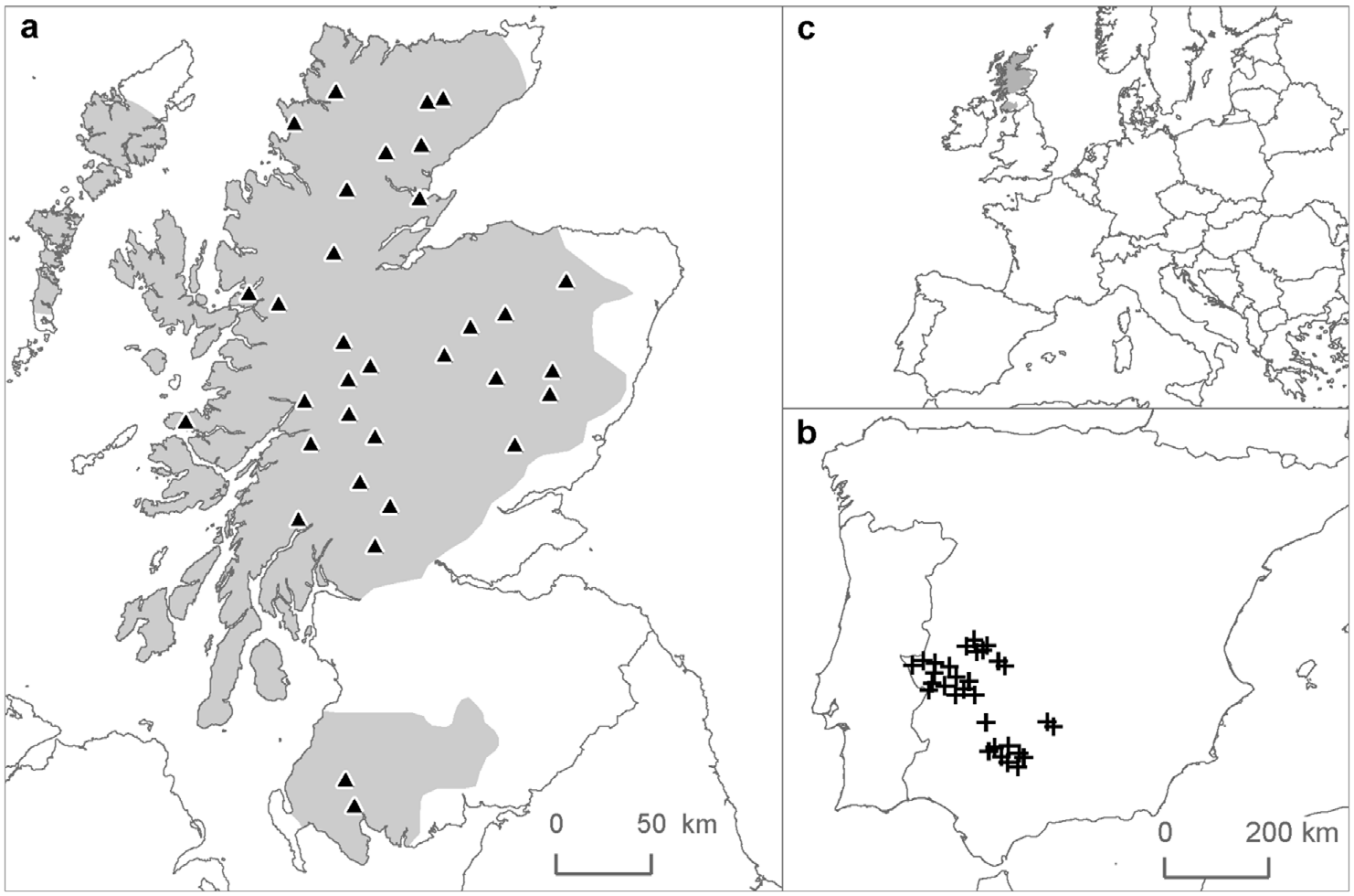
Sampling sites, (a) Scotland, (b) Spain, (c) locations within Europe. The actual distribution of red deer within each population is shaded in grey. The number of symbols to locate red deer subpopulations in Spain has been reduced to improve readability but they are a good representation of the range of the study area.

We used samples of 3323 (1674 females, 1649 males) Scottish red deer shot in 36 locations and 7020 (2664 females, 4356 males) Iberian red deer shot in 63 locations in Southern Spain between 1997 and 2011 (Fig 1). The deer were aged between 1 and 17 years old (1^st^ quartile = 2, 3^rd^ quartile = 6, mean = 5.4). All the samples came from legal game activities of private estates in Scotland and Spain or from culling operations of the Forestry Commission for Scotland. The animals were shot from a distance using a high power marksman rifle fitted with a riflescope. No animals were killed specifically for this study. The mandible bones were extracted by technicians or stalkers, at the shooting site or in the associated game larder, and were stored in a freezer or left outdoors for the flesh to decompose, until they were collected and transported to the laboratory. Shooting date, sex, and location (at estate level) were also recorded for each animal.

**Fig 2:**
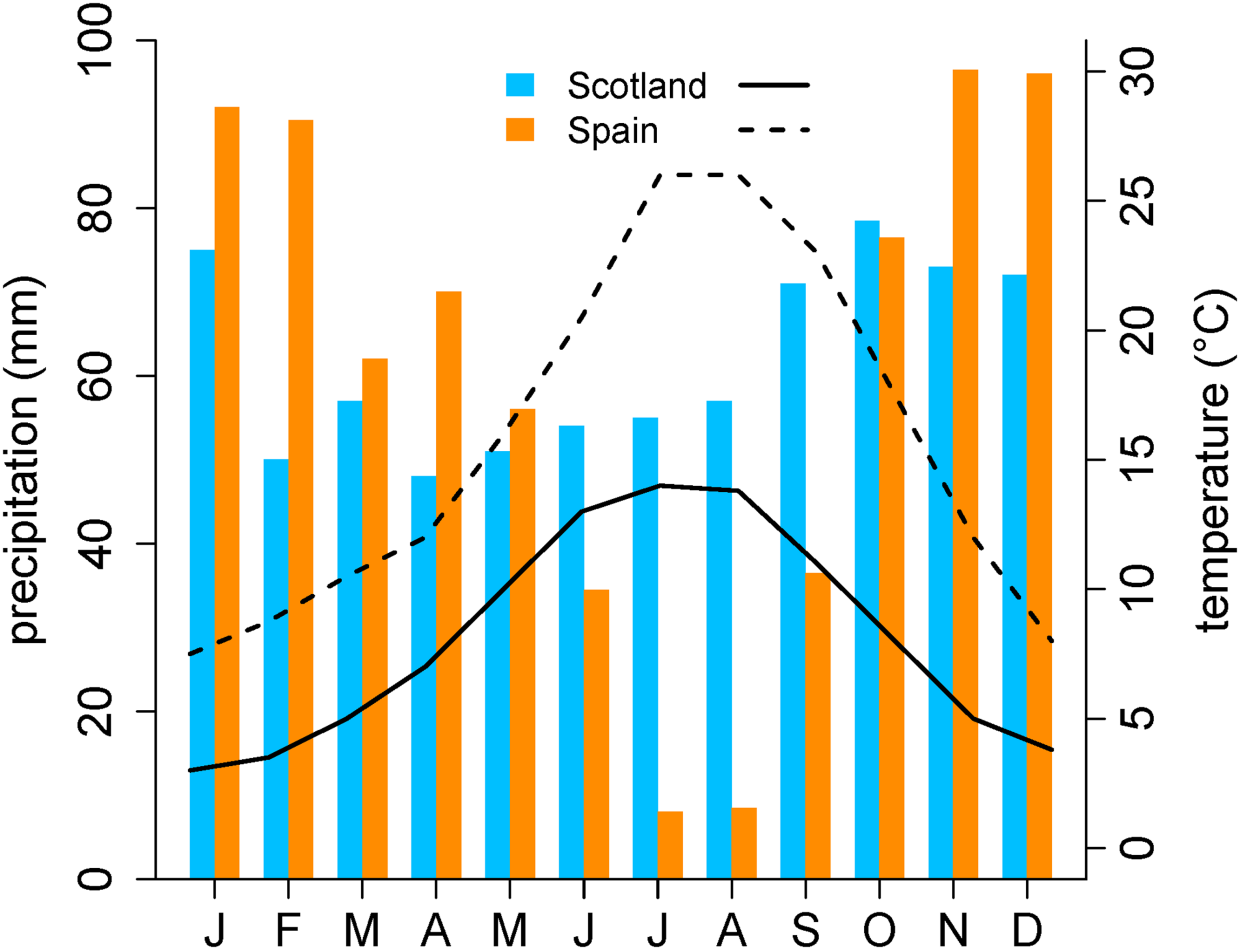
Monthly mean temperature (lines) and precipitation (bars) in the two study areas, Scotland 1971–2000 and Spain 1961–1990. Data source: MetOffice (http://www.metoffice.gov.uk/) and Nuñez Corchero & Sosa Cardo [65].

Red deer ranges in Scotland and Spain differ strongly in ecological conditions. The Iberian climate is much hotter and drier in summer with a strong seasonal variation in both temperature and precipitation in comparison with the Scottish red deer range (Fig 2). In Scotland, the predominant habitats within red deer range are grasslands, heaths, peatland and forestry plantations, while in the Spanish study area there are less grasslands and forest plantations and a predominance of scrublands and oak open woodlands (dehesas) (S1 Table). The contrasting seasonality and habitats produce remarkable differences in diet selection between populations, favouring more grazing in Scotland and comparatively more use of browse in Spain (S2 Table).

### Mandible length, age and tooth wear

In the laboratory, the length of the mandible (ML, ± 1 mm) was measured as a proxy of skeletal size (i.e. from the mesial border of the first incisor socket to the vertical part of the ramus, after having removed the flesh in these two points).

To estimate the age and tooth wear, mandibles were sectioned through a frontal plane between the entoconid-hypoconid and metaconid-protoconid of the first molar (M_1_) using a circular diamond saw [26]. The age (in years) was estimated by counting the milky-coloured cement layers on the root pad of the sectioned mesial section of M_1_, aided by a reflected light microscope at magnification x20 to x25 [27,28]. When M_1_ was missing, or the cement layers poorly differentiated, M_2_ was used and the age in years was estimated as the number of cement layers plus one.

Tooth wear was estimated by measuring, with the aid of a calliper and a magnifying glass, the thickness of the dentine on the sectioned mesial section of M_1_ (molar height, MH, ± 0.1 mm) from the top of the cementum of the radicular pad to the middle point of the sectioned crown [7,26]. It has been noted that although crown formation in M_1_ is fully complete at the age of 4 months [19,29], the completion of eruption and final positioning of the molar in the mandible does not take place until 3 years of age in red deer [19,20], and teeth also move in the mandible at very old age. Consequently, measuring molar height perpendicular from the mandible bone, labial or buccal, is not a reliable measurement of molar wear, especially in young age classes; by contrast, our MH measurement is independent of any movement of the molar in the mandible. We also measured crown size of the first permanent incisor (incisor height, IH, ± 0.1 mm), from the labial gingival sulcus to the median sagittal plane top of the crown [1].

Tooth wear was assessed as the negative relationship of crown height with age [7,26].

### Statistical analysis

Lifespan for each sex and population was calculated as the age at the 99^th^ quantile of the frequency distribution of the age of the samples.

We used linear mixed-effects regression models to predict the response of MH, IH and ML against a number of predictors, controlling for some sources of variation. Normality and homoscedasticity were verified. Inspection of plots of fitted values against residuals [30] revealed some clear outliers, probably due to typing errors during data acquisition and inaccurate age estimation of some animals, and these were removed from the data.

The full linear mixed-effects model on MH and IH attempted to fit two additive random effects (the intercepts of location and year of shooting) and three fixed effects: age, sex and population (Scotland—Spain) as well as their meaningful first and second order interactions. Age was fitted as a quadratic term, rather than using non-linear or splines approaches, as linear models are preferred when testing for interactions [31], and because we wanted to keep the model terms simple so that interactions were manageable. In addition we included ML as a covariate in the fixed effects (the best estimator of body size, condition independent, that we had available), in order to ensure that molar height and incisor height were independent of body size in the analysis, and therefore appropriate proxies of molar and incisor wear, respectively. ML was only retained in those models where it was statistically significant.

Two additional mixed models, one to assess changes in rates of tooth wear between MH and IH, and the other to assess the effect of MH on ML, were carried out by fitting the same random effects described above, along with the fixed effects of quadratic age, sex and population, together with their first and second order interactions.

Despite the recent controversy surrounding the use of p-values against measures such as ΔAIC or BIC, their use has been clarified recently [32]. As our objective was to identify the main drivers of our dependent variables, rather than create predictive models, we correctly used p-values to define our final models in favour of ΔAIC or BIC approaches. We proceeded by first fitting full models, as described above, and then using backward elimination we removed the non-significant fixed-effects terms, one at a time, following the principle of marginality: the highest order interactions were tested first and if they were significant, then the lower order effects were not tested for significance.

The coefficients of the final model were calculated using REML [33]. As in linear mixed-effects models, determining the correct value of degrees of freedom in the estimate of the coefficients is meaningless [30,33], we used Satterthwaite’s degrees of freedom approximation. The variance explained by the model was represented as *R* ^*2*^ marginal (variance accounted for by the fixed effects; *R* ^*2*^_LMM(m)_) and *R* ^*2*^ conditional (variance accounted for by random and fixed effects; *R* ^*2*^_LMM(c)_), following a method developed for linear mixed-effects models [33]. All analyses and graphics were conducted in R software (R Development Core Team 2012), mainly using lme4 [34] and lmerTest [35] packages.

## Results

### Lifespan

Males and females of the Scottish population were more longevous (male = 14 yrs, female = 15 yrs) than those of the Iberian population (male = 10 yrs, female = 14 yrs). Sexual differences in lifespan in favour of females were higher in the Iberian population (4 yrs) than in the Scottish one (1 yr).

### Molar and incisor wear: sex and populations

**Fig 3:**
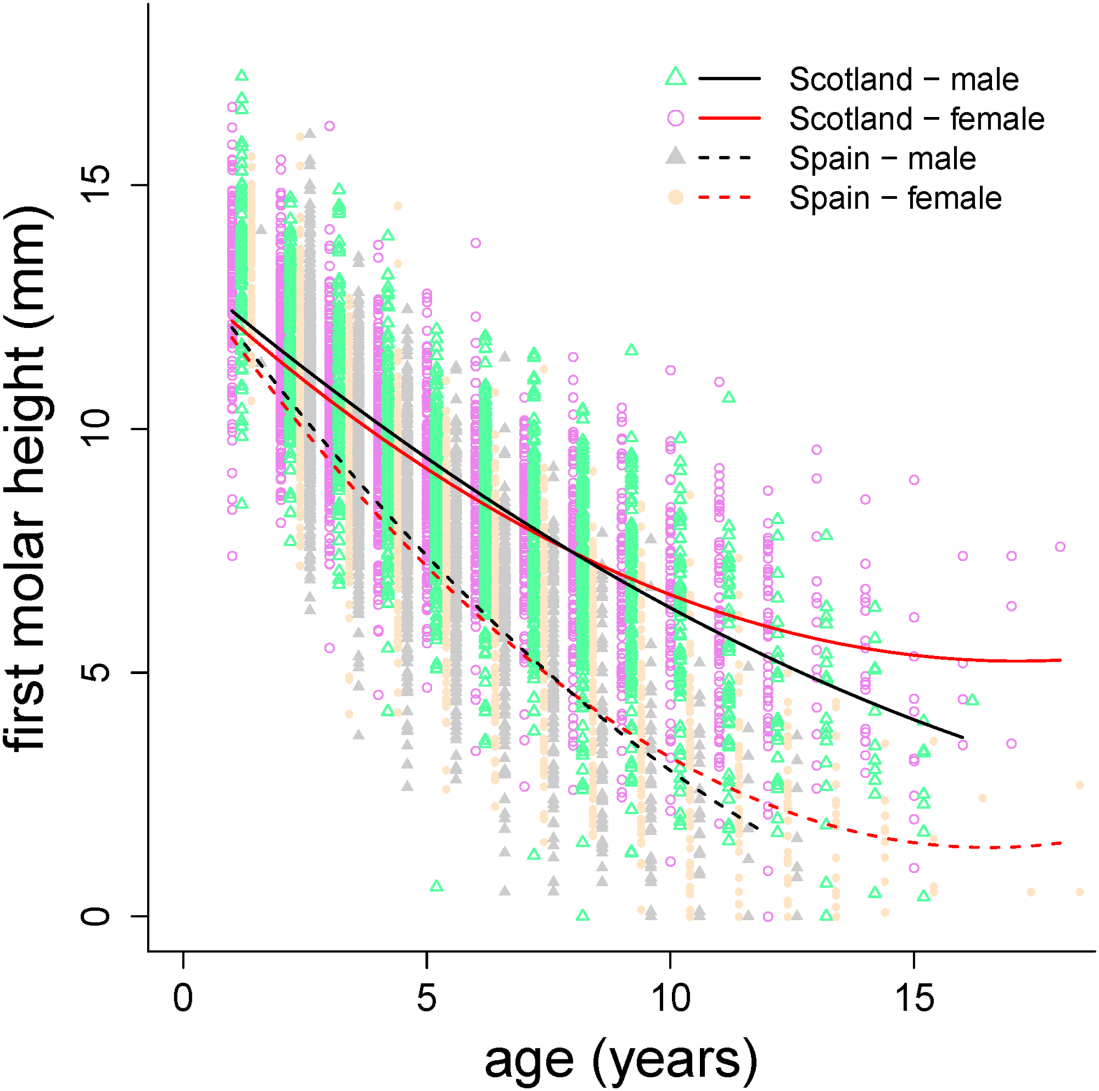
Predictions of first molar height against age for animals of mean mandible length of the model in Table 1 for males and females red deer of Scottish and Iberian (Spain) populations.

The final model on MH included location and year as additive random effects and, as fixed effects, ML, age^2^, sex, population and the interactions age^2^ × sex, and age^2^ × population (Table 1). The fixed effects explained 68.0% of the variance of the data (*R* ^2^_LMM(m)_, Table 1) and the total variance explained by fixed and random effects was 73.5% (*R* ^2^_LMM(c)_, Table 1). MH was similar in both populations at the age of 1 year old (p = 0.517), but for age classes older than 1 year old it was smaller in Iberian than in Scottish red deer (p < 0.0001, for each age class), with a remarkably increased rate of tooth wear for males and females in the Iberian populations in comparison with the Scottish ones (Table 1, Fig 3). Rates of molar wear up to 7.5 years old were similar between sexes, but as age increased further the rates of molar wear in males were higher than in females in both populations. As an example, the predicted average depletions of M_1_ (taking the predicted MH at the age of 1 year old by sex as the reference) for 11 year old individuals, were 53.2% and 48.9% for Scottish males and females, and 80.9% and 76.9% for Iberian males and females, respectively (Table 1, Fig 3).

**Table 1:** Coefficients of the linear mixed-effects model on the height of the first molar (an index of tooth wear, mm). *R* ^2^_LMM(m)_: *R* ^2^ marginal (variance account for the fixed effects); *R* ^2^_LMM(c)_: *R* ^2^ conditional (variance account for random and fixed effects). Age in years; Age^2^: quadratic term of age; ML: mandible length (cm).

Random effects				
Groups	Variance	std dev		
location (intercept)	0.3154	0.5616		
year (intercept)	0.1188	0.3447		
Residual	2.0923	1.4465		
Fixed effects				
	Estimate	std error	t-value	p
intercept	17.806	0.4502	39.550	<0.0001
ML	-0.181	0.0179	-10.070	<0.0001
age	-0.919	0.0401	-22.910	<0.0001
age^2^	0.027	0.0029	9.310	<0.0001
sex (male)	0.143	0.1294	1.110	0.269
population (Spain)	0.156	0.1888	0.830	0.408
age x sex (male)	0.072	0.0477	1.500	0.133
age^2^ x sex (male)	-0.011	0.0038	-3.000	0.003
age x population (Spain)	-0.515	0.0493	-10.460	<0.0001
age^2^ x population (Spain)	0.017	0.0038	4.370	<0.0001
R^2^_LMM(m)_	0.6802			
R^2^_LMM(c)_	0.7351			

**Fig 4:**
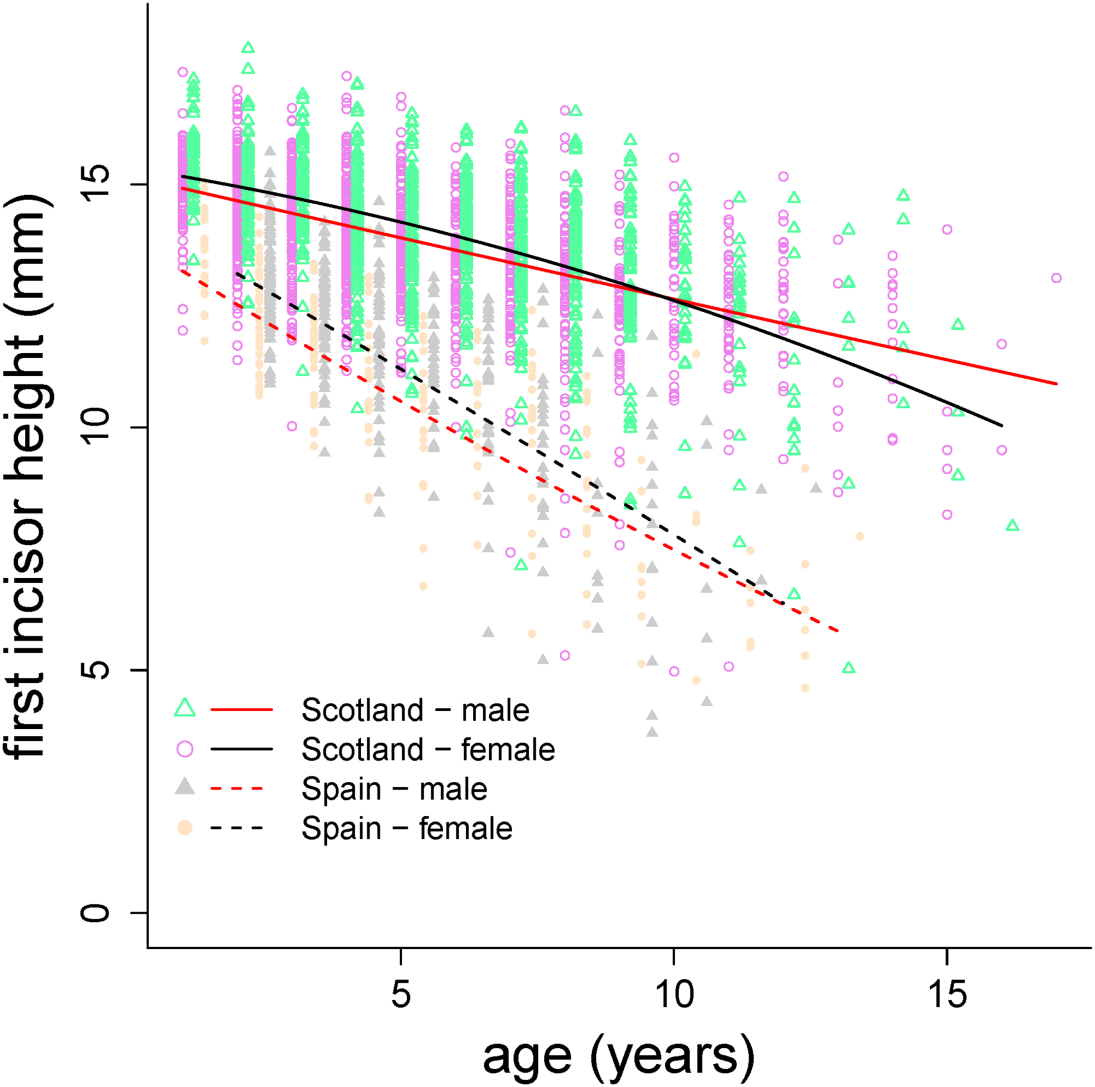
As Fig 3 but predictions are for incisor height using the model in Table 2.

The terms in the final regression model for IH were the same as those in the MH model above, with the exception that the covariate ML was not included, as it was not significant, and there was one additional interaction, sex × population (Table 2). The fixed effects explained 57.3% of the variance of the data (*R* ^2^_LMM(m)_, Table 2) and the total variance explained by fixed and random effects was 63.4% (*R* ^2^_LMM(c)_, Table 2). Both males and females of Iberian red deer had smaller IH than Scottish red deer, and that was true across all age classes. At yearling, Iberian red deer males had higher predicted IH than females of the same population, however in Scottish red deer yearling males and females had similar IH (Table 2, Fig 4). Iberian populations had faster rates of incisor wear than Scottish populations in males and females, and within population males wore their incisors at a faster rate than females did. As an example, the predicted average depletions of I_1_ at an age of 11 years old, were 19.4% and 17.0% for Scottish males and females, and 48.7% and 47.7% for Iberian males and females, respectively (Table 2, Fig 4).

**Table 2:** Coefficients of the linear mixed-effects model on the height of the first incisor (an index of tooth wear, mm). Acronyms as in Table 1.

Random effects				
Groups	Variance	std dev		
location (intercept)	0.15898	0.3987		
year (intercept)	0.07165	0.2677		
Residual	1.39122	1.1795		
Fixed effects				
	Estimate	std error	t-value	p
intercept	15.176	0.1630	93.090	<0.0001
age	-0.256	0.0359	-7.150	<0.0001
age^2^	0.000	0.0027	0.100	0.9208
sex (male)	0.178	0.1554	1.140	0.2532
population (Spain)	-1.253	0.2493	-5.030	<0.0001
age x sex (male)	0.079	0.0530	1.490	0.1357
age^2^ x sex (male)	-0.010	0.0040	-2.470	0.0135
age x population (Spain)	-0.455	0.0789	-5.760	<0.0001
age^2^ x population (Spain)	0.006	0.0066	0.960	0.3353
sex (male) x population (Spain)	0.338	0.1256	2.690	0.0071
R^2^_LMM(m)_	0.5727			
R^2^_LMM(c)_	0.6335			

**Fig 5:**
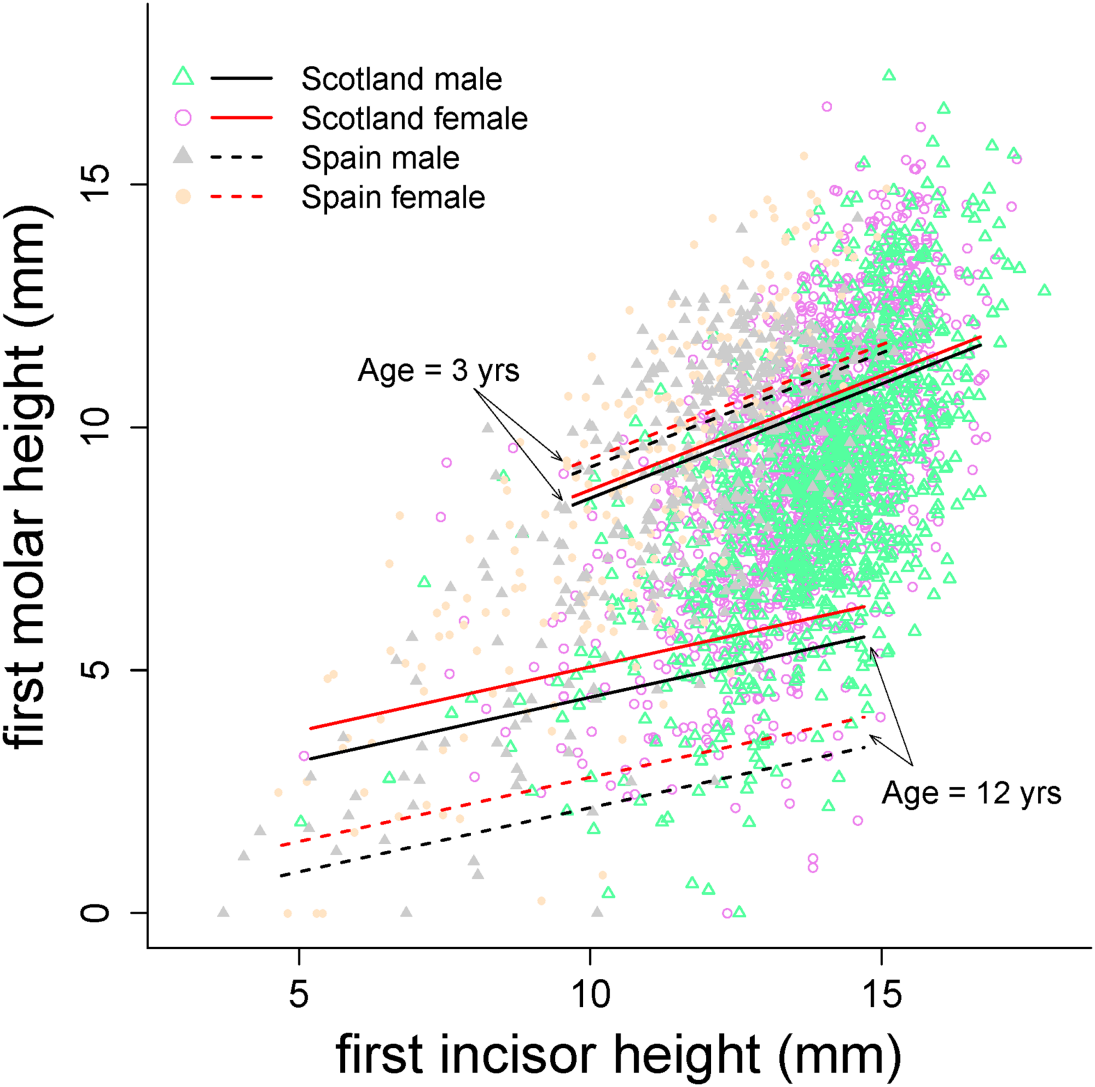
Predictions of molar height against incisor height of the model in Table 3 for males and females red deer of Iberian and Scottish populations at 3 and 12 years old.

The model to assess the relationship of tooth wear rate between MH and IH, used MH as the response variable, included location and year as additive random effects, and the fixed effects age^2^, sex, population and IH together with the interactions age^2^ x sex, age^2^ x population and age^2^ x IH (Table 3). The fixed effects explained 69.9% of the variance of the data (*R* ^2^_LMM(m)_, Table 3) and the total variance explained by fixed and random effects was 72.6% (*R* ^2^_LMM(c)_, Table 3). There was a positive correlation between MH and IH across all age classes, that did not interact with sex or population, which suggests that the relationship in the pattern of wear between molars and incisors was maintained across sexes and populations. However, the positive correlation between MH and IH was linearly weaker as animals of both sexes and populations got older, as indicated by the interaction between age and IH (estimate of linear effect = -0.051, se = 0.0189; p = 0.007, Table 3, Fig 5).

**Table 3:** Coefficients of the linear mixed-effects model on the height of the first first molar (mm) against the height of the first incisor (IH, mm). Acronyms as in Table 1.

Random effects				
Groups	Variance	std dev		
location (intercept)	0.1476	0.3842		
year (intercept)	0.0486	0.2205		
Residual	2.0219	1.4219		
Fixed effects				
	Estimate	std error	t-value	p
intercept	4.533	1.0150	4.465	<0.0001
age	-0.176	0.2672	-0.658	0.511
age^2^	0.0001	0.0169	0.001	0.999
sex (male)	-0.442	0.1833	-2.410	0.016
population (Spain)	1.509	0.3057	4.938	<0.0001
IH	0.606	0.0687	8.822	<0.0001
age x sex (male)	0.126	0.0642	1.965	0.050
age^2^ x sex (male)	-0.012	0.0049	-2.410	0.016
age x population (Spain)	-0.282	0.1012	-2.786	0.005
age^2^ x population (Spain)	-0.003	0.0086	-0.324	0.746
age x IH	-0.051	0.0189	-2.695	0.007
age^2^ x IH	0.002	0.0013	1.488	0.137
R^2^_LMM(m)_	0.6992			
R^2^_LMM(c)_	0.7258			

### Mandible length and molar wear

**Fig 6:**
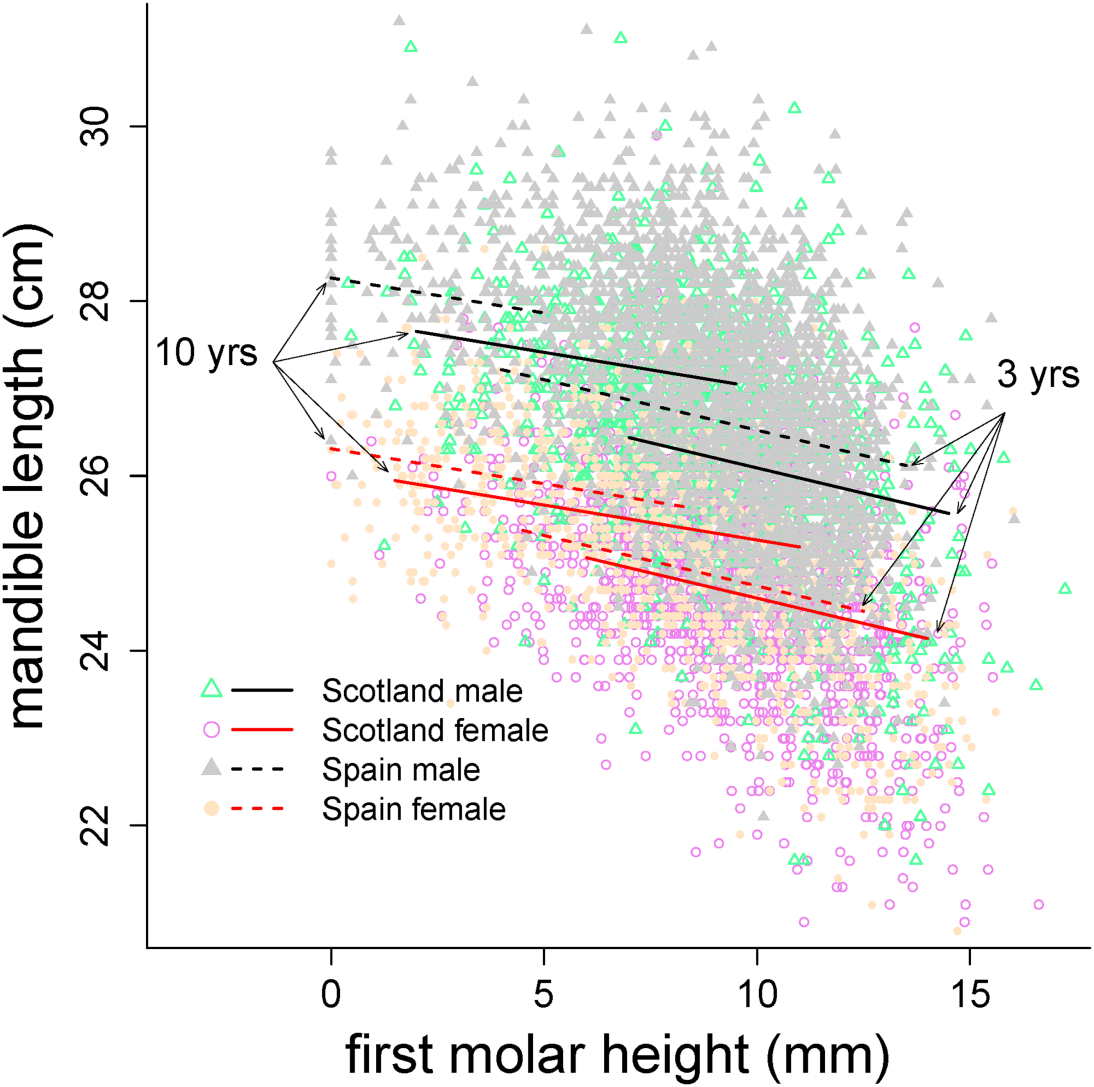
Predictions of Iberian and Scottish red deer mandible length against first molar height of the model in Table 4 in animals of 3 and 10 years old. IB, circle, dashed line: Iberian population; SC, triangle, solid line: Scottish population; Black line: male; grey line: female. Thick line: prediction at age = 10 yrs; Thin line: prediction at age = 3 yrs.

ML responded to the fixed effects of age^2^, sex, population, MH, and the interactions age^2^ × MH, population × MH, and age^2^ × sex × population (Table 4, Fig 6). The fixed effects explained 53.1% of the variance of the data (*R* ^2^_LMM(m)_, Table 4), and the total variance explained by fixed and random effects was 65.7% (*R* ^2^_LMM(c)_, Table 4). Males had longer mandibles than females, and mandibles of the Iberian deer were longer than those of Scottish deer (Table 4, Fig 6). Although the response was not strong, shorter MH (i.e. more worn M_1_) in both sexes and populations was significantly related to longer mandibles across age classes (Table 4, Fig 6).

**Table 4:** Coefficients of the linear mixed-effects model on the mandible length against the height of the first molar (MH, mm). Acronyms as in Table 1.

Random effects				
Groups	Variance	std dev		
location (intercept)	0.3085	0.5555		
year (intercept)	0.0074	0.0861		
Residual	0.8593	0.9270		
Fixed effects				
	Estimate	std error	t-value	p
intercept	26.458	0.3045	86.890	<0.0001
age	-0.315	0.0578	-5.440	<0.0001
age^2^	0.028	0.0036	7.790	<0.0001
sex (male)	0.879	0.2489	3.530	<0.0001
population (Spain)	-0.602	0.2802	-2.150	0.032
MH	-0.308	0.0216	-14.270	<0.0001
age x sex (male)	0.251	0.0466	5.390	<0.0001
age^2^ x sex (male)	-0.016	0.0032	-5.090	<0.0001
age x population (Spain)	0.287	0.0465	6.180	<0.0001
age^2^ x population (Spain)	-0.021	0.0033	-6.370	<0.0001
age x MH	0.082	0.0050	16.420	<0.0001
age^2^ x MH	-0.006	0.0004	-14.550	<0.0001
sex (male) x population (Spain)	0.717	0.1753	4.090	<0.0001
sex (male) x MH	-0.012	0.0154	-0.760	0.450
population (Spain) x MH	0.052	0.0164	3.190	0.001
age x sex (male) x population (Spain)	-0.181	0.0659	-2.750	0.006
age^2^ x sex (male) x population (Spain)	0.013	0.0054	2.430	0.015
R^2^_LMM(m)_	0.5308			
R^2^_LMM(c)_	0.6569			

## Discussion

Our results put forward contrasting patterns of tooth wear between the two red deer populations, as well as between the sexes and type of teeth (molars, incisors), and for the incisor size at emergence, and pose new questions on the causes of these differences during the evolutionary divergence of the lineages.

In particular, the results of this study indicate that (i) the rates of molar and incisor wear were much higher in the Iberian population than in the Scottish one (as predicted in H_1_); (ii) at yearling age, Scottish red deer had larger incisor crowns than Iberian red deer, as also did males compared to females only for the Iberian population (partially in agreement with ideas implicit in prediction H_2_), but molar height was similar between populations and sexes (against prediction H_2_); (iii) males depleted their teeth earlier than females in both populations (in agreement with prediction H_3_); (iv) molar wear rate was higher than incisor wear rate (as predicted in H_4_), especially in the Iberian population; but while molar wear was curvilinear and decelerated with age in both sexes, incisor height decreased more linearly with age, with only a slight accelerating pattern in males; (v) longer mandibles associated with more worn molars in both sexes evidencing a trade-off between body growth and preserving molar teeth (as predicted by the first statement in H_5_), which did not differ between populations (against the second part of prediction in H_5_).

### Interpopulational differences in tooth wear

The main finding of this study was the big difference between populations in the patterns of tooth wear, both molar and incisor, which wore faster in Spain than in Scotland. The environmental and population conditions experienced by Iberian and Scottish red deer are extremely contrasting (see Methods). In addition, in Scotland, red deer densities varied between 0.5 and 42.6 deer/km^2^ across a period of 43 years (1961–2004) and 128 estates, with mean and median values of 8.3 and 8.1 deer/km^2^, respectively, and 95% of the density records were between 1.7 and 23.2 deer/km^2^ [36]. By contrast, in Spain, deer densities across the study area were on average four times higher (mean = 33.9, median = 35, min = 11.0, max = 46.0, 95% of the records between 11.0 and 43.9 deer/km^2^). These differences in deer density, along with the increased climatic seasonality and use of abrasive xeric Mediterranean vegetation (See S2 Table and references herein), might increase the constraints on food energy density and the associated dental wear in the Spanish population. Our results also showed that the factor ‘location’ made the greatest contribution of the random effects on molar and incisor wear, indicating that conditions prevailing at each particular area (e.g. hunting estates) can play an important role in tooth wear, in agreement with previous interpopulation comparisons [18,37,38].

### Sex differences in tooth wear

Sexual selection may have affected molar and incisor wear in both populations. Our results show that molar wear decelerated more in females than in males in older ages and, as a consequence, teeth were depleted earlier in males than in females (Figs 3 and 4); in males some accelerating pattern of incisor wear also took place. Sex differences in tooth wear might appear either because of differences in diet selection [22] or in the optimal timing of tooth use relative to the sex-specific timing of growth and reproduction across lifetime [7,16]. However, differences in diet would be expected to produce sex differences in wear across all ages; the fact that most differences found increasingly manifest after prime age suggests a predominant role of the optimal timing of tooth use, which in this case produced differences between males and females, thus pointing to sexual selection as a relevant cause of our findings. This is in agreement with previous work on molar wear in this species [5,7,16]. However, note that the smaller effect of the interaction between sex and age found here, in comparison with that same effect found in Spanish populations [7], is not comparable as the datasets and the statistical methods are different. But on the other hand, the effect of sex across age on tooth wear appears to be variable among populations. For example, no sex difference was found in the red deer of Rum [39] and Veiberg et al. [20] reported high interpopulational variation in molar and incisor wear for red deer in Norway.

Kubo et al. [38] studied two sika deer (*Cervus nippon*) populations and only found heavier incisor wear in males in comparison with females in the population with stronger wear. These results suggest that although sexual selection may ultimately be responsible for male and female differences in tooth wear, environmental conditions may act to modulate them as has been shown for many other sexually dimorphic traits (see Bondurianski [40] and references herein).

### Wear patterns in molars and incisors

Molars and incisors have different functions, these being comminution and cropping, respectively. We found that molar wear decelerated with age, especially in females, while incisor wear was linear in females and slightly accelerating in males. We may speculate that the energy requirements of males for sexual competition after prime age might cause a more rapid depletion of teeth [7], and this would explain the accelerating wear observed in incisors. In the case of molars, this decelerating pattern has been already observed in previous studies [5,7,16,26,39]. In these studies, including the present one, molar wear was estimated only in the first lower molar M_1_. Unlike incisors, comminution by M_1_ can be displaced to other molars as they erupt, thus reducing workload per unit of occlusal surface area (see e.g. Ozaki et al.[37]). Even so, however, the deceleration in wear rate was lower in males than in females evidencing that males were less able to cope with their energy needs and maintain molar durability as were females [16,21].

The scaling between molar and incisor height was similar in males and females in both populations (Table 3; Fig 5), which suggests that incisor and molar teeth functions are linked in the same way in both sexes and populations and across age.

The negative relationship between molar wear and our proxy of body size (mandible length), but not between incisor wear and mandible length suggests that molar function (comminution efficacy) is more closely related to body growth than incisor function (cropping) [2,9,20]. Additionally, molar wear may not have noticeable consequences for fitness until molar functionality is severely affected [7,39], as the occlusal surface of the molar can remain operative across a wide range of molar wear [2,5,9]. In contrast, for incisors, crown size, incisor breadth and protrusion influence bite size [10,41,42], with the result that wear may have direct consequences on intake by cropping activity, although we are unaware of any study on the effects of incisor wear in fitness.

### The evolution of hypsodonty and lifespan

In the present study, molar teeth were found to emerge at similar size regardless of population and sex, which is not in agreement with the hypothesis (H_2_) that heavy wear may favour the evolution of increased hypsodonty. Further, the observed difference in the size of incisors at emergence between populations is contrary to H_2_ prediction of more hypsodont incisors in populations subjected to higher rates of tooth wear. However, for the population with greater incisor wear rate (Spain), we found that male incisors were more hypsodont at emergence than female ones.

These results raise the question of why increased hypsodonty has not evolved as a general feature of teeth in the population and sex with greater tooth wear. The hypothesis that high levels of tooth wear should lead to hypsodonty is based on the assumption that selection will act to maintain tooth durability and lifespan [17,43]. However, it is already known that this is not the case when comparing molar morphology between males and females in red deer [16], and in other body size dimorphic ungulates [21], when longer lifespan in males does not translate into longer reproductive lifespan [44] and hence does not have fitness returns. However, the lack of difference in molar hypsodonty between populations with very different wear rate, together with the finding that incisors are in fact less hypsodont in the population (Iberian) with heavier wear, deserves further explanation.

We found considerable differences in longevity between populations (ca. 4 years), with longevity especially shorter in males from the population with the higher rate of tooth wear (Iberian red deer), which suggests that tooth wear could be a proximal cause of these differences. These results for differences in longevity might be spurious, however, if caused by human mediated management conditions or bias in our sampling methods. Previous studies using samples from hunting activity have shown that, although culling may affect the sample size of different age classes, it has little effect on the relationship between the variables measured and age; therefore reliable information can be acquired on the relationships between wear patterns, longevity, senescence and sex differences [16,45]. However, we cannot rule out that current management conditions favouring high densities in Spain may play a role in promoting heavy tooth wear. Indeed, the effect of location in our analyses of MH and IH indicates the relevance of differences in local conditions for tooth wear.

Regardless, differences in wear and longevity between Scottish and Spanish red deer are likely caused by contrasting environmental conditions, although we are unaware to what extent current conditions have been operating across evolutionary time in both populations. The differentiation of Iberian and Scottish red deer probably took place with the last deglaciation period in the early Holocene (11700 yrs BP) [23,24,46]. Although extinctions of Arctotertiary woody taxa took place during the Early and Middle Pleistocene, glacial refugia in coastal shelves of the Mediterranean and intramountainous valleys facilitated the survival of a number of temperate, Mediterranean and Ibero-North African woody angiosperms [47]. Palaeobotanical analysis of the Pleistocene floras and vegetation in the Iberian Peninsula shows the existence of patchy landscapes with *Pinus* woodlands, deciduous and mixed forests, parklands (savannahlike), shrublands, steppes and grasslands [47]. This suggests that the ancestors of current Iberian and Scottish red deer might have adapted, during the Pleistocene at the southern refugia, to environments that included Mediterranean floras. Scottish deer ancestors left the Iberian refugia and colonised land, exposed by the retreating ice, in the north, which was probably dominated by cryptogams and herbaceous species which were not as tough as the vegetation in the south. However, climate has changed during the Holocene in all Western Europe, resulting not only in the deglaciation of northern areas, but also an increase in temperatures and xerification events in southern ranges [48,49], which might have led to an increase in the traits of resistance to physical damage present in many Mediterranean plants and responsible for higher rates of tooth wear in deer. Thus, it is difficult to say whether Scottish deer ancestors have shifted from an environmental scenario with a dental structure adapted to a more abrasive vegetation to a scenario with a softer vegetation, making their dentition comparatively more durable in the new environment, or whether Spanish deer ancestors have experienced the opposite process during the Holocene in Iberia. Indeed, both changes may have occurred simultaneously and jointly contribute to the differentiation in tooth wear between lineages. Veiberg et al. [20] found for red deer in Norway that populations with the smallest molars tended to wear teeth more rapidly, suggesting that maximum tooth height may be limited by the same factors that lead to rapid wear. On the other hand, Kubo & Yamada [50] compared populations of sika deer and found measurable differentiation in molar hypsodonty only for phylogenetic lineages that had diverged for more than 0.3 Mya, suggesting that such a minimum time might be required for evolution of hypsodonty. In addition, populations expanding north from Iberian refugia may have been selected for a dispersal phenotype (bigger size [46]) while the remaining, Southernmost Iberian populations may have experienced the strongest and most persistent increase in temperatures and xerification events [48], thus leading to a maintenance phenotype, economically smaller and shorter living, associated with heavier rates of tooth wear, when foraging conditions became more extreme than in the environment in which the species originally evolved. This scenario may not correspond with data of current body size due to changes during the Holocene. A recent study on Norwegian red deer shows a reduction in body size related to changes in the environment mediated by humans, such as landscape fragmentation, increase of grazing domestic animals and hunting pressure [51]. This situation may have independently affected both Spanish and Scottish populations making it difficult to interpret our results.

Selection is expected to produce optimal solutions for trade-offs between reproduction and maintenance for longevity [52]. Cost and benefits along the gradient of variation in the solutions for a trade-off are most relevant when compared with those adopted by individuals within the same population [53–57];together with responses based on phenotypic plasticity and reaction norms [58–60] this may lead to higher than initially expected differences between populations that followed independent evolutionary pathways in different environments. In other words, if populations that moved from Iberia to Northern Europe experienced lower wear and higher longevity as a result of e.g. softer vegetation, we should not necessarily expect that a new selective force should act on Iberian deer to increase their longevity, despite the fact that we can now see an evident difference between populations.

Differences in hypsodonty found for incisors, however, require another explanation. While the main negative effect of molar wear on fitness may not occur until the ages of severe depletion [39], incisor size is instrumental in determining arcade size and consequently the bite size and intake [10,41,42]. This effect may be relevant in the evolution of sex differences in Iberian red deer, if larger males compensate for increased wear in Spain, and also between populations, if either body size or diet differed between populations during evolution. Data on current diets suggest a higher proportion of grasses and forbs in the diet of Scottish deer, compared to Iberian red deer that appear to feed more on ligneous vegetation (see S2 Table and references herein). Grazing would select for large incisor-arcade breadth for cropping efficiency, while browsing is characterised by a more selective feeding style that favours narrow arcades [13,61,62]. The possible relationship of mandible and incisor size with the type of diet and the mode of cropping during evolution since the divergence of both populations from the last glacial maximum deserves further investigation.

Further research including biometric information from common ancestors at the last glacial maximum in the Iberian refugia, as well as in the following stages during the divergence of both lineages, would help to clarify the processes causing the phenotypic divergence between both lineages and the relative roles of past and present conditions in shaping the current contrasting patterns of tooth wear and life history.

## Supporting Information

S1 Table Main features of the land cover of the study area. Mosaics features of Scotland have been incorporated into their predominant single feature. Sources: MLURI [63] and Observatorio de la Sostenibilidad en Espana [64].
(DOCX)

S2 Table Diet composition of red deer in Scotland and Spain. Data have been averaged across seasons when available. Values of an index of browsing in curly brackets. The browsing index is the percentage of individual plants browsed with respect to the total individuals (n = 25646) of the plant species found in 544 transects of 50 m x 2 m during the months of June and July 2004. Sources: [66–69].
(DOCX)
