# Supplementary material for: Wear Fast, Die Young: More Worn Teeth and Shorter Lives in Iberian Compared to Scottish Red Deer
Source: PLoS One. 2015 Aug 7;10(8):e0134788. doi: 10.1371/journal.pone.0134788 (PMC4529110; doi:10.1371/journal.pone.0134788)
Supplement: S1 Table — Mosaics features of Scotland have been incorporated into their predominant single feature. Sources: MLURI [63] and Observatorio de la Sostenibilidad en Espana [64]. (DOCX) [file pone.0134788.s001.docx]

# Supporting Information

S1 Table

|  | % cover |  |
| --- | --- | --- |
| Cover types | Scotland | Spain |
| **Open land** |  |  |
| Arable | 11.2 | 31.0 |
| grassland | 23.2 | 15.5 |
| bracken | 1.4 |  |
| heather moorland | 15.8 |  |
| peatland | 24.6 |  |
| montane | 3.7 | 0.2 |
| rock and cliffs | 0.4 |  |
| **woodland** |  |  |
| plantations | 12.2 | 6.4 |
| mixed woodland | 1.1 | 0.1 |
| broadleaved | 1.3 | 1.3 |
| scrub | 0.1 | 18.6 |
| Dehesa |  | 24.5 |
| **wet ground** |  |  |
| fresh waters | 1.9 | 1.7 |
| marshed | 0.2 |  |
| saltmarshes | 0.1 |  |
| dunes | 0.2 |  |
| **developed** |  |  |
| rural + urban | 2.4 | 0.7 |
