# Supplementary material for: Wear Fast, Die Young: More Worn Teeth and Shorter Lives in Iberian Compared to Scottish Red Deer
Source: PLoS One. 2015 Aug 7;10(8):e0134788. doi: 10.1371/journal.pone.0134788 (PMC4529110; doi:10.1371/journal.pone.0134788)
Supplement: S2 Table — Data have been averaged across seasons when available. Values of an index of browsing in curly brackets. The browsing index is the percentage of individual plants browsed with respect to the total individuals (n = 25646) of the plant species found in 544 transects of 50 m x 2 m during the months of June and July 2004. Sources: [66–69]. (DOCX) [file pone.0134788.s002.docx]

# Supporting Information

S2 Table

|  | % in diet |  |  |
| --- | --- | --- | --- |
| species | Scotland | Spain | source |
| *Cistus ladanifer* |  | 20 | [66] |
| *Cistus populifolius* |  | {65} | Unpublished |
| *Cistus salvifolius* |  | {37} | Unpublished |
| *Fraximus excelsior* |  | 2 | [66] |
| *Myrtus communis* |  | {56} | Unpublished |
| *Olea europaea* |  | 2, {72} | [66], Unpublised |
| *Phillyrea angustifolia* |  | {65} | Unpublished |
| *Phillyrea latifolia* |  | {65} | Unpublished |
| *Quercus coccifera* |  | {45} | Unpublished |
| *Quercus rotundifolia* |  | {50}, 11 | Unpublished [66] |
| *Quercus suber* |  | 27 | [66] |
| *Rubus ulmifolius* |  | 16 | [66] |
| grasses | 69, 45, 85, 55 | 27 | [66], [67], [68], [69] |
| forbs | 0.5, 2, 15 |  | [67], [68], [69] |
| dwarf shrubs *(Ericacea* mainly *Calluna vulgaris*) | 26, 50, 14, 31 |  | [67], [68], [69] |
| others (unclassified) | 4, 4, 2 |  | [67], [68], [69] |
| Source: Unpublished: Torres-Porras and Carranza. | | | |
